# Realization of High-Reliable Coherent-State Quantum Secure Communication

**DOI:** 10.34133/research.1290

**Published:** 2026-06-01

**Authors:** Xinlei Chen, Geng Chai, Lei Wang, Sijie Wang, Xiaojie Chen, Zhengwen Cao

**Affiliations:** Laboratory of Quantum Information and Technology, School of Electronic Information, Northwest University, Xi’an 710127, China.

## Abstract

Continuous-variable quantum secure communication encoded by Gaussian mapping offers high-capacity and high transmission rates. For the theoretically secure encryption scheme of a 1-time pad, a highly reliable coherent-state quantum secure communication system has been established, and its security has been quantitatively evaluated using Wyner’s wiretap channel theory. We also propose an information reconstruction scheme based on multidimensional rotation to extract secret messages at low-to-medium signal-to-noise ratios. Meanwhile, to address the unbalanced optical path, we design a self-balanced homodyne detector based on a programmable gain amplifier, achieving an electronic noise variance of $1.624\times 10^{-7}$ $V^{2}$ and a bandwidth of 715 MHz. In 10-km optical fiber transmission, the system successfully achieved secure transmission of the dichroic image, processed 1,536 information blocks, each containing $2^{10}$ continuous variables, with a block error rate of approximately $8.78\times 10^{-5}$, and ultimately achieved the secrecy capacity of $2.44\times 10^{5}$ bits per second.

## Introduction

As we transition from the era of traditional computing to the new era of quantum computing, numerous vital cybersecurity issues must be reconsidered. The 1-time pad is an encryption technique that cannot be cracked in cryptography, and its key is a single-use preshared key [1]. However, with advances in theory and technology, quantum computers can bypass existing defense mechanisms with their formidable computing power, completely disrupting or cracking the digital encryption systems that modern information and communication infrastructure rely on [2,3].

To address the security threats posed by brute-force key-cracking, quantum communication provides protection against eavesdropping and computational cracking based on the principles of uncertainty, measurement collapse, and the noncloning theorem in quantum mechanics [4–6]. The groundbreaking BB84 quantum key distribution (QKD) protocol established an essential milestone in quantum communication [7], which marks an essential milestone in quantum communication. Since then, quantum communication technology has continued to develop and some practical schemes have been applied in information security. For example, QKD has been used to ensure the security of information transmission [7,8]; quantum teleportation enables long-distance transmission of information without the need for direct transmission of physical carriers [9]; quantum secure communication (QSC) [10], also known as quantum secure direct communication, is different from QKD, which transmits random numbers over a quantum channel. QSC provides a direct method for the legitimate communication parties to transmit secret messages through the quantum channel [10,11]. There are 2 primary methods for establishing the quantum correlation between communication parties in QSC. The first is the 2-step protocol based on dense coding, which uses Einstein–Podolsky–Rosen pairs, whose theoretical security depends on error-free transmission of the checking sequence, and also identifies the secure transmission conditions under noisy channels [11]. Subsequently, the team used random single-photon bit encoding to propose another scheme without entanglement sources, namely the DL04 protocol, which can ensure its security [12].

QSC emerged after QKD, but numerous theoretical protocols and applications have been developed over the past 2 decades. QSC has gradually become a research hotspot in QSC [13]. Typical advances include the use of the Greenberger–Horne–Zeilinger state [14], multiuser QSC networks [15,16], 1-step QSC [17], passive-state preparation QSC [18], decoy-state QSC [19,20], receiver-device-independent QSC [21], QSC based on quantum error correction code [22], and semantic QSC [23]. At the same time, measurement-device-independent protocols and device-independent protocols have also been proposed, laying a solid foundation for using actual devices for QSC in communication [24–26]. In terms of experiments, the early experiments based on the single-photon platform have confirmed that QSC can operate under noise and loss [27]. Entanglement-based QSC using quantum memory has been experimentally demonstrated [28], including a 500-m implementation based on optical fiber entanglement sources [29]. These pioneering experiments realized early QSC protocols and established a solid foundation for subsequent technological development. Sun et al. [30] employed delay encoding to replace quantum memory, thereby addressing the bottleneck posed by the lack of practical quantum memory. Subsequently, the integration of classical coding theory with QSC was developed to tackle the security challenges of high loss and code errors [31], with the security analysis completed using Wyner’s wiretap channel theory and the first practical system being developed [32,33]. These technological advances enabled QSC to utilize optical fibers for intercity communication, reaching a maximum distance of 100 km [34]. Additionally, experimental demonstrations of QSC on optical fiber channels have been achieved without active polarization compensation [35].

In addition to optical fiber communication solutions, QSC based on single photons has been implemented in free-space channels [36]. Furthermore, QSC has been combined with postquantum cryptography to construct secure repeaters, enabling the establishment of large-scale quantum communication networks while mitigating the security risks associated with trusted repeaters [37]. Recently, a key breakthrough was achieved in a 1-way quasi-QSC protocol with single photons, demonstrating stable transmission at 2.38 kilobits per second over 104.5 km of optical fiber [38], with a 4,760-fold rate increase compared to 2022 [34]. In network scenarios, a QSC scheme employing a dual-pumped structure successfully achieved 300-km QSC among 4 pairs of users [39].

The QSC technology mentioned above is based on discrete variable systems. Due to its early research and rapid development, the related demonstration models and experiments have matured quickly. In contrast, continuous variable (CV) protocols are compatible with classical electro-optical communication devices and transmit continuous symbols, thereby achieving higher communication capacity and faster communication rates. The first CV quantum secure communication (CV QSC) protocol employed coherent states as light sources [40]. Since then, various CV QSC protocols based on single-mode and entangled states have emerged [41–44]. Among them, the Gaussian mapping (GM) provides a convenient and efficient modulation method to achieve accurate Gaussian modulation. Moreover, the feasibility and effectiveness of the 2-step CV QSC based on GM have been experimentally verified in the optical fiber channel, and the corresponding parameter estimation scheme is established [43]. In addition, a 20 megabits-per-second high-speed quantum radio-frequency-over-light communication based on CV quantum dense coding with entangled states has been experimentally realized [45]. Recent studies have shown that the deterministic purification scheme applied to the coherent-state system can effectively suppress excess noise [46], and QSC experiments with squeezed states have also been demonstrated [47]. Furthermore, a highly reliable QSC based on the concatenation of Gottesman–Kitaev–Preskill codes and quantum low-density parity-check codes has been developed [48].

Although protocols based on CV entanglement sources are feasible, their system complexity is considerably higher than that of single-mode implementations. In this context, we develop an experimental platform for coherent-state QSC based on a 1-time-pad scheme. The main contributions of this work are summarized as follows:

• This study successfully achieved highly reliable and secure transmission of the dichroic image in a 10-km standard single-mode fiber channel by building a coherent-state QSC with a 1-time pad experimental system, verifying the feasibility of the established system in actual communication scenarios.
• The accurate transmission of secret messages relies on the strong correlation between the statistical characteristics (SC) of Gaussian variables shared by the communicating parties. However, as the transmission distance increases or channel conditions degrade, significant discrepancies in SC may arise, leading to reduced accuracy in message recovery at the receiver. To address this issue, we propose an information reconstruction scheme based on a multidimensional rotation model, which includes both direct and indirect reconstruction modes. The core idea is to enable all parties to mutually recover Gaussian variables, reconstruct their statistical properties, and accurately extract secret messages through multidimensional rotation and inverse operations.
• During the detection stage, we observe that unbalanced optical paths introduce additional common-mode noise, which severely degrades system performance. To address this issue, we design and implemented a self-balanced homodyne detector (self-BHD) based on a programmable gain amplifier. The proposed self-BHD enables automatic balancing at high bandwidth without requiring any modification to the optical path or the introduction of additional optical components. Ultimately, an electronic noise variance of $1.624\times 10^{-7}$ $V^{2}$ was achieved, with a bandwidth of 715 MHz.
• Finally, on the basis of the proposed information reconstruction and the designed self-BHD, an efficient coherent-state QSC system was successfully implemented. In this system, the original image is encoded in 1,536 information blocks after GM, each containing $2^{10}$ CV quantum states. The experimental results indicate that the system achieved a secrecy capacity of $2.44\times 10^{5}$ bits per second under the condition of a 10-km fiber channel, with a block error rate of about $8.78\times 10^{-5}$, substantially improving image quality and demonstrating the practical value of this system in quantum communication applications.

The paper is structured as follows. Results and Discussion first introduces the system setup, followed by simulation analysis, and then verifies the system performance through image transmission experiments. Conclusion summarizes the above results. Methods presents the information reconstruction scheme in detail and provides a quantitative evaluation of the system security.

## Results and Discussion

### System implementation

**Fig. 1.**
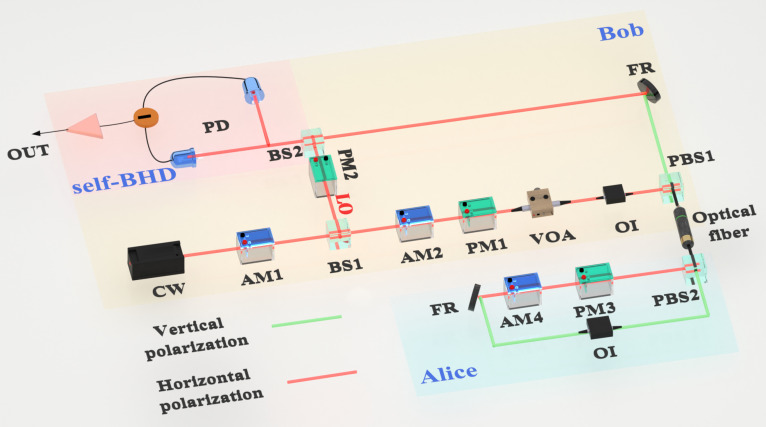
Experiment setup. CW, 1,550-nm continuous-wave light source; BS, beam splitter; AM, amplitude modulator; PM, phase modulator; VOA, variable optical attenuator; OI, optical isolator; PBS, polarization beam splitter; FR, Faraday rotator; PD, photodiode; self-BHD, self-balanced homodyne detector; LO, local oscillator. Alice and Bob are in the same laboratory and separated by a 10-km optical fiber spool.

The experimental setup for the proposed system is shown in Fig. 1. On Bob’s side, coherent light pulses with 100 ns and a repetition frequency of 1 MHz are generated by a 1,550-nm laser diode and an amplitude modulator (AM1) and divided into weak signal light and strong local oscillator (LO) light using an extremely unbalanced beam splitter (BS1) [49]. The 2 quadratures of the signal light are modulated by another amplitude modulator (AM2) and the phase modulator (PM1) to obtain the Gaussian distribution. The signal pulse is attenuated to the quantum level by a variable optical attenuator and then sent to Alice via a 10-km optical fiber channel. The optical isolator allows the signal light to enter only the secret-message modulation module via 1 port of the polarization beam splitter (PBS2). At Alice’s side, the modulation of the secret messages is accomplished using an amplitude modulator (AM4) and a phase modulator (PM3). After being reflected by the Faraday rotator, the polarization direction of the light rotates $90^{\circ }$, changing from horizontal to vertical polarization. Thus, it can be ensured that the polarization state emerging from PBS2 must be vertical and then transmitted to Bob through the same channel. Similarly, PBS1 and optical isolator ensure that the pulses transmitted from Alice are output only through ports aligned with their polarization. Subsequently, Faraday rotator restores the signal pulse’s polarization direction to its initial state for measurement. At the beam splitter BS6, the signal pulse interferes with the LO light. Among these components, the phase modulator (PM4) modulates the LO path phase to select the measured quadrature components.

Because interference is highly sensitive to phase difference, random phase drift in the signal light disrupts stable interference with the LO light, thereby affecting the demodulation of secret messages. To this end, phase correction can be achieved by alternating transmission of quantum signal and classical reference pulses. In the specific implementation, odd-order pulses are used as signal carriers to carry Gaussian-modulated quantum information. Even-order pulses are used as phase references and without phase changes [46]. Meanwhile, by regulating the voltages of the electro-optic modulators AM2 and PM1, the reference pulse amplitude is increased to approximately 500 times the mean amplitude of the signal pulse. Finally, the self-BHD extracts the modulation information of the signal light.

**Coherent-state QSC protocol:** The basic communication protocol involves 2 parties, Alice, the sender of secret messages, and Bob, the receiver. The goal is to enable Alice to send secret messages to Bob based on the Gaussian random numbers transmitted by Bob. Therefore, Alice and Bob need Gaussian modulators, and the forward and backward channels refer to the 2 opposite transmission directions of a physical channel [46,50]. The specific process of the protocol is as follows:

1. Bob generates 2 sets of Gaussian random numbers $G_{1}$ and $G_{2}$ with zero mean and variance $V_{B}$ to modulate the quadrature position *x* and momentum *p* of coherent states. He sends the modulated quantum states to Alice through the forward quantum channel.
2. The 2 Gaussian sequences received by Alice can be represented as $t_{1}G_{1}$ and $t_{1}G_{2}$ (channel excess noise is not considered for easy understanding), where $t_{1}$ is a parameter determined by the transmittance of the forward channel. Alice uses a BS to split the received coherent-state light into 2 beams, 1 for eavesdropping detection. Secret messages are transmitted using another beam when no eavesdropping is detected.
3. After determining channel security, Alice maps the secret message to 2 Gaussian random sequences $M_{1}$ and $M_{2}$ with zero mean and variance $V_{A}$ through GM (see Supplementary Materials Section S1.1 for details) and then modulates the light beam that transmits the secret message using these 2 sequences. The modulated beam is transmitted back to Bob through the backward channel, which can be given by $t_{1}t_{2}G_{1}+t_{2}M_{1}$ and $t_{1}t_{2}G_{2}+t_{2}M_{2}$. $t_{2}$ is a parameter determined by the backward channel transmittance.
4. Bob employs heterodyne detection to measure both quadrature components simultaneously. Under ideal detection conditions, the parameters $t_{1}$ and $t_{2}$ are estimated to derive the Gaussian random sequences $M_{1}$ and $M_{2}$. Through inverse GM, Bob can successfully decode the secret messages originally encoded by Alice from $M_{1}$ and $M_{2}$.

### Simulation results analysis

This section will simulate the system performance of direct and indirect schemes based on security analysis (see the “System security analysis” section for details). The classic error-correcting code selected is the polar code, constructed based on channel polarization [51]. Currently, only this coding technique can theoretically be proven to achieve Shannon capacity and has linear complexity. Due to the need to construct an orthogonal matrix, the maximum dimension of multidimensional rotation is 8 dimensions *d* = 8. The simulation is set to include $2^{15}$ subinformation blocks per information block, for a total of 10^7^ information blocks. Gaussian variables are divided into 8 equal-probability intervals. The other parameters in the simulation are as follows: modulation variance *V_B* = 5 SNU, *V_A* = 8 SNU, excess noise *ϵ* is 0.01; the standard loss of single-mode optical fiber cable is 0.2 dB/km.


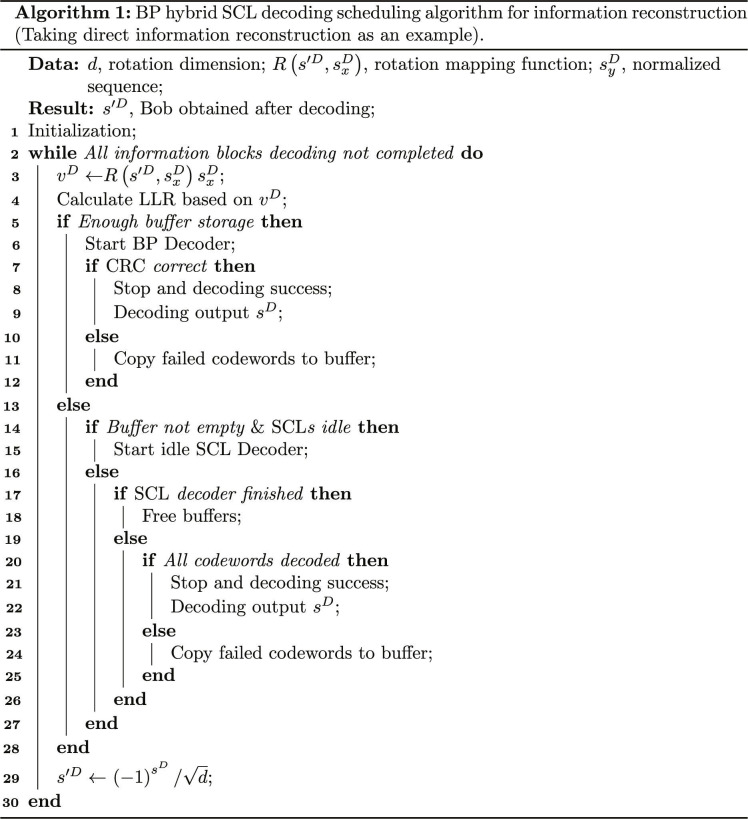


The information reconstruction of this scheme involves a complex decoding process. With numerous secret messages and long code lengths, substantial computational resources are needed for encoding and decoding, potentially causing delays and difficulties in reconstruction. Algorithm optimization and hardware improvements are essential to address these issues. We adopt a general processing unitaccelerated hybrid decoding [52] approach combining belief propagation (BP) [53] and successive cancellation list (SCL) decoding [54]. Although SCL decoding offers superior bit error rate performance for polar codes by leveraging sequential node correlations, it lacks parallel processing capabilities. In contrast, BP decoding enables efficient parallelization. This hybrid mechanism (Algorithm 1) synergistically integrates both methods to achieve accuracy and efficiency. Taking direct information reconstruction as an example, Bob calculates the log-likelihood ratio (LLR) of the received information

$$\mathrm{LLR}=\frac{2\left\|M_{2}\right\|\left\|M_{2}^{\prime }\right\|v^{D}}{\sqrt{d}\sigma^{2}}$$ (1)

When BP decoding fails the cyclic redundancy check after maximum iterations, the codeword is forwarded to a parallel SCL decoder. The computational overhead has been reduced by implementing BP and SCL decoding on the general processing unit. The system’s throughput can be approximated as

$$T_{\mathrm{hyb}}=\frac{T_{\mathrm{BP}}\cdot T_{\mathrm{SCL}}}{{\text{IBER}}_{\mathrm{BP}}\cdot T_{\mathrm{BP}}+T_{\mathrm{SCL}}}$$ (2)

where *T*_BP_ is the time required for BP decoding, including the number of iterations and the duration of each iteration. *T*_SCL_ is the time required for the SCL decoding, which is only executed when the BP decoding fails. IBER_BP_ is the information block error rate for BP decoding.

**Fig. 2.**
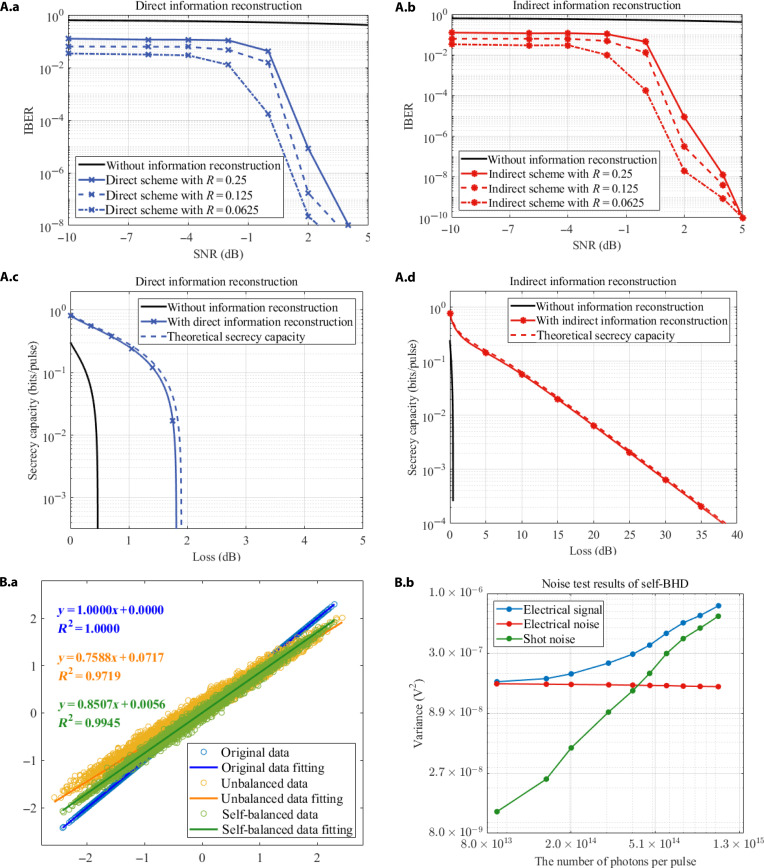
(A) Comparison of information block error rate (IBER) and secrecy capacity with and without information reconstruction’s coherent-state quantum secure communication (QSC) system under different signal-to-noise ratio (SNR) environments. (A.a) The IBER of direct information reconstruction. (A.b) The IBER of indirect information reconstruction. The horizontal axis represents the SNR, which ranges from −10 to 5 dB, and the vertical axis represents IBER. (A.c) Secrecy capacity of direct information reconstruction. (A.d) Secrecy capacity of indirect information reconstruction. The horizontal axis represents the channel loss, and the vertical axis represents the secrecy capacity. The black solid line represents the case without information reconstruction, the blue curve with crosses represents the application of direct reconstruction, the red curve with stars represents the application of indirect reconstruction, and the different line shapes represent different polar code rates *Rc* = 0.25, *Rc* = 0.125, and *Rc* = 0.0625. The dashed lines represent the theoretical secrecy capacity curves of the proposed scheme under different information reconstruction schemes. (B) The test results of relevant indicators of the self-balanced homodyne detector (self-BHD). (B.a) Statistical distribution of data under self-balanced and unbalanced optical paths. (B.b) Electrical noise test results of self-BHD.

Figure 2A.a and A.b compare IBER with and without information reconstruction under different signal-to-noise ratio (SNR) environments. It can be seen that without information reconstruction (solid black line), the IBER is around $10^{-1}$, and when the SNR is below 0 dB, the IBER exceeds 0.5. This result indicates that, under low-to-medium SNR conditions, extracting secret messages without information reconstruction is poor. However, the curves for direct (blue curve with crosses) and indirect (red curve with stars) information reconstruction are always lower than those without information reconstruction, indicating a significant improvement in information extraction accuracy. Especially when the SNR approaches 5 dB, IBER reduces by at least $10^{7}$ times compared to without information reconstruction, and the minimum is around $10^{-8}$. This marked improvement confirms that the proposed information reconstruction can enhance IBER performance in coherent-state QSC, enabling the system to extract more secret messages with lower IBER at low-to-medium SNR, meeting the needs of high reliability and secure communication. In addition, Fig. 2A.a and A.b also reveal a trend: As the code rate decreases, IBER also decreases, indicating that the information reconstruction effect is more ideal under low-code-rate conditions. However, low code rates often affect the system’s overall decoding efficiency. Therefore, the most suitable code rate should be selected based on specific environments and requirements to achieve optimal system performance in practical applications.

Figure 2A.c and A.d simulate and analyze the secrecy capacity with and without information reconstruction under different channel losses. The dashed lines represent the theoretical secrecy capacity curve under different information reconstruction schemes when IBER=0. By comparison, as the channel loss increases, the black curve representing information reconstruction without is always lower than the curve without information reconstruction, which approaches 0. Meanwhile, the performance of information reconstruction is close to its theoretical secrecy capacity. This indicates that information reconstruction greatly improves secrecy capacity and exhibits greater tolerance to channel loss, which is expected to play a key role in long-distance communication applications of CV QSC systems. Furthermore, by comparing Fig. 2A.c and Fig. 2A.d, it can be observed that the indirect outperforms the direct in terms of secrecy capacity and channel loss tolerance, while the direct scheme is relatively simple. Therefore, a suitable reconstruction scheme can be selected based on different practical application environments to achieve optimal performance and implementation efficiency.

### Experimental verification and analysis

This section conducts experimental verification of the proposed scheme based on the experimental setup shown in Fig. 1 and analyzes the experimental results. As the system’s key component, the modulation and detection of quantum signals directly determine the system’s performance. At the end of modulation, we adopt Gaussian modulation. This technology has the advantage of high information capacity and maintains good compatibility with classical communication systems. The module has recently been optimized, enabling the modulation of the quantum signal through adaptive photoelectric calibration in an field-programmable gate array-based miniaturized unit [55]. During the detection stage, we observed that an unbalanced optical path would introduce additional common-mode noise, substantially affecting system performance. Thus, we designed a self-BHD to compensate for the unbalanced optical path and then applied it to the experimental platform. The designed self-BHD is detailed in the Supplementary Materials Section S1.2. This self-BHD achieves high-bandwidth automatic balance control without modifying the optical path or adding optical devices. It should be noted that existing methods involve the addition of variable optical attenuators to achieve self-balance, but such solutions would reduce the detection efficiency of the system [56].

Firstly, we tested and compared the output signals in a balanced and an unbalanced optical path to evaluate the detector’s performance. In short, enable the self-balanced function, record the output signal after the optical path is balanced, then turn off the function, and manually set the programmable-gain amplifier gain to the same value to simulate an unbalanced optical path. The test results are shown in Fig. 2B.a. The self-balanced (green fitting line) not only exhibits better linear characteristics than the unbalanced (yellow) but also markedly improves agreement with the initial signal (blue). The test results for the variance of the output electrical signal, the electrical noise, and the shot noise of the detector under different LO intensities are shown in Fig. 2B.c. When the input LO intensity of the self-BHD is 128 μW, the number of photons per pulse is $10^{15}$, and the shot noise is about 6.7 dB higher than the electrical noise. In addition, the self-BHD has the following characteristics: The electronic noise variance is $1.624\times 10^{-7}$ $V^{2}$, the bandwidth is about 715 MHz, with a gain fluctuation in the passband less than 3 dB, and the common-mode rejection ratio of the self-BHD is 59.77 dBm. Please refer to the illustration in the Supplementary Materials Section S1.2 for specific test results.

**Fig. 3.**
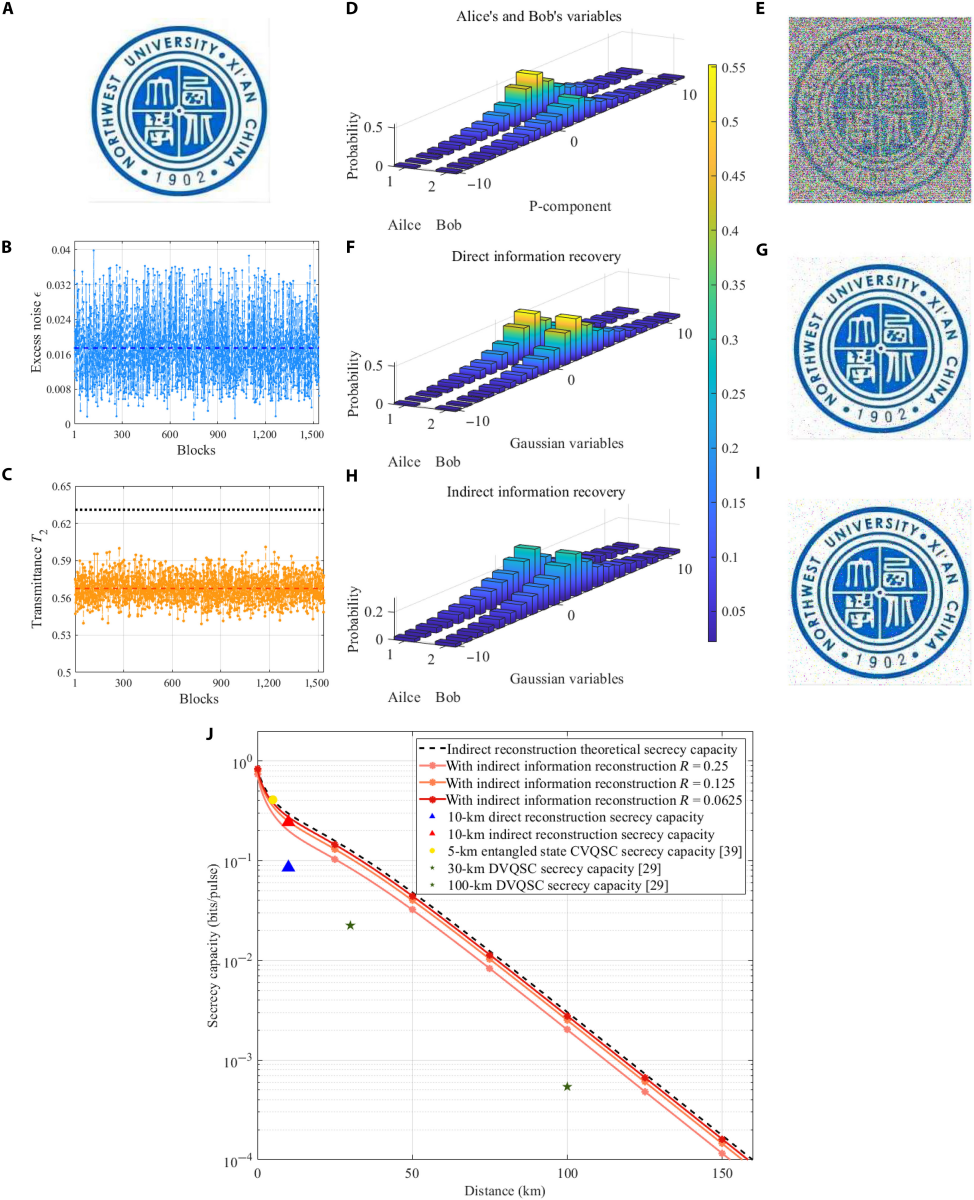
Experimental results. (A) Original image. (B) Excess noise results. (C) Channel transmittance results. The small solid circles in (B) and (C) represent the results of these 1,536 information blocks as the objects of observation. The dashed line represents the average value of all information blocks. The dashed line between points represents the data’s volatility. The black dotted line represents the theoretical upper bound of channel transmittance. (D) SC of P-component Gaussian variables for Alice and Bob. The horizontal axis represents Gaussian variables, and the vertical axis represents probability. (E) The restored image without information reconstruction. (F) Gaussian variables of both communication parties after direct information reconstruction. (G) The restored image after direct information reconstruction. (H) Gaussian variables of both communication parties after indirect information reconstruction. (I) The restored image after indirect information reconstruction. (J) The relationship between the transmission distance and the secrecy capacity.

To demonstrate the effectiveness of this scheme more intuitively, we selected the dichroic image shown in Fig. 3A. In the experiment, 1,536 information blocks were transmitted, each containing 2^10^ variables. To simplify the measurement system’s complexity, we measure only 1 quadrature component, randomize it, perform symbol mapping, and then apply spherical mapping to both the original and symbol-mapped sequences. The number of intervals divided is 8, the multidimensional rotation dimension is *d* = 8, and the polar code rate is *R_c* = 0.125. All experimental data were decoded on an NVIDIA GeForce RTX 2080 Ti. To ensure the reliability of the results, each information block is independently decoded $10^{4}$ times. Experimental performance of 10-km optical fiber transmission is shown in Table 1, which also compares the error rates of the existing systems [43,45] with our system. Our system can achieve an error rate of $8.78\times 10^{-5}$ at 1.39 dB, indicating a greater information extraction advantage than previous systems. Figure 3B and C present the experimental results of excess noise and channel transmittance for 1,536 information blocks in the backward channel. The results indicate that the fluctuation range is relatively small, with mean values of 0.017 (SNU) and 0.568, respectively, confirming the experimental system has good stability.

**Table 1.** Overview of experimental performance of 10-km optical fiber transmission. SNU represents the unit of shot-noise units. *V_A_*, Alice’s modulation variance; *ϵ*, excess noise; *T*_2_, the backward channel transmittance; IBER, information block error rate; SC, secrecy capacity; bps, bit per second; *T_hyb_*, throughput of the hybrid decoder; SNR, signal-to-noise ratio.

Parameter	*V_A_* (SNU)	*ϵ* (SNU)	*T*_2_	IBER	SC	*T_hyb_*
Value	10	0.017	0.568	$8.78\times 10^{-5}$	$2.44\times 10^{5}$ bps	38.52 Mbit/s
	Cao et al. [43]		Liang et al. [45]		Our work	
SNR (dB)	1.89		11∼12		1.39	
Error rate	10^−3^		$\left(3.0\pm 0.9\right)\times 10^{-4}$		$8.78\times 10^{-5}$	

Figure 3D shows the SC of Gaussian variables of P-component in the backward channel between Alice and Bob, indicating the validity of the experiment’s measured data and providing a data basis for subsequent information reconstruction steps. Fig. 3E is the restored image without information reconstruction corresponding to Fig. 3A. After direct information reconstruction, the SC of Alice and Bob’s Gaussian variables are shown in Fig. 3F, and the corresponding restored images are shown in Fig. 3G. Figure 3H and I, respectively, show the SC of Gaussian variables and the restored images after indirect information reconstruction. By comparing these restored images with the original images, we can observe that information reconstruction shows clear advantages compared to without information reconstruction, with substantial improvements in image accuracy and clarity. These results further validate the effectiveness of the proposed information reconstruction, indicating that it can effectively enhance the system’s reliability and anti-interference ability. The relationship between the transmission distance and the secrecy capacity is shown in in Fig. 3J. The yellow circle represents the results based on entangled state CV QSC in a 5-km optical fiber [43]. The star markings represent the results of discrete variable QSC under 30- and 100-km optical fiber transmission, respectively [34]. The triangle corresponds to the results obtained by the system using both direct and indirect information reconstruction modes in a 10-km optical fiber. The dashed line represents the theoretical secrecy capacity for indirect information reconstruction, and the solid line with stars represents the secrecy capacity at different code rates (this relationship is derived based on the calibration parameters in Table 1). It is worth noting that all experimental data points for the compared systems lie below this system’s curve, highlighting its marked advantage in improving confidentiality capacity and demonstrating its application prospects in long-distance communication.

## Conclusion

This work establishes a coherent-state QSC experimental platform and achieves reliable image transmission. Among them, the original image is encoded in 1,536 information blocks after GM, each containing $2^{10}$ CV quantum states. The system achieved a secrecy capacity of $2.44\times 10^{5}$ bits per second under a 10-km fiber channel, fully verifying the feasibility and practicality of the proposed scheme. Specifically, we propose an information reconstruction scheme based on a multidimensional rotation mathematical model to solve the problem of poor accuracy in extracting secret messages under low-to-medium SNR, which includes 2 methods: direct and indirect reconstruction, and the lower limit of secrecy capacity for these 2 schemes was calculated separately based on Wyner’s wiretap channel theory. Subsequently, the results show that information reconstruction performs excellently at low-to-medium SNRs (−10 to 5 dB) and can efficiently extract secret messages. Compared to without information reconstruction, IBER reduces up to 10^7^ times, which improves the accuracy of secret messages. Furthermore, in response to the issue of an unbalanced optical path, we have also designed a self-BHD based on a programmable gain amplifier. The test results show that the electronic noise variance of the detector is $1.624\times 10^{-7}$ *V*^2^, and the bandwidth is about 715 MHz. Finally, the established experimental platform verified the coherent-state QSC system with information reconstruction and self-BHD. The results show that this system exhibits excellent image transmission performance and achieves an IBER of about $8.78\times 10^{-5}$. This achievement strongly confirms the effectiveness and applicability of this information reconstruction mechanism in practical systems.

The information reconstruction scheme proposed in this study effectively improves the transmission reliability of the coherent-state QSC system. However, in the scheme, transmitting additional information through the classical channel still incurs communication resource occupation, which limits the system’s efficiency to some extent. In response to this technical bottleneck, subsequent research will focus on breaking through the dependence on classical channels and will be committed to developing an efficient information reconstruction mechanism based on quantum channels. The breakthrough in this direction is expected to substantially enhance the practical value of the CV QSC system and promote its engineering process.

## Methods

### Information reconstruction for coherent-state QSC

Secret messages can be transmitted through Gaussian modulation using GM [43]. However, unknown channel characteristics and imperfect devices inevitably affect quantum state transmission and measurement. These factors lead to considerable discrepancies between the Gaussian variables SC of Alice’s encoded messages and Bob’s received signals. Although the GM interval has a certain tolerance for noise, as this deviation increases, the system’s tolerance will gradually decrease, affecting the accuracy of secret messages.

An information reconstruction scheme is proposed to address the above issues. Alice divided the secret message into *M* information blocks and performed uniformization into subinformation blocks through GM (GM rules are based on precommunication agreements) to obtain Gaussian variables *M*_1_ and *M*_2_. Alice uses *M*_1_ and *M*_2_ to modulate the 2 quadrature components that transmit the secret messages. The modulated output beam is sent to Bob through the backward channel. After measuring and removing the effect of his previously modulated information, Bob holds 2 sets of Gaussian variables $M_{1^{\prime }}$ and $M_{2^{\prime }}$ that are related but inconsistent with the sequences $M_{1}$ and $M_{2}$. The quantum channel involved is simplified into a standard linear model to describe the correlation between Alice and Bob’s data, namely: $M_{1^{\prime }}=t_{2}M_{1}+Z$, $t_{2}=\sqrt{\eta T_{2}}$, $T_{2}$ is the backward channel transmittance, η is the detector efficiency, *Z* is the total Gaussian noise in the backward channel, satisfying a Gaussian distribution $N\left(0,\;\sigma^{2}\right)$, and $\sigma^{2}$ is the noise variance.

**Fig. 4.**
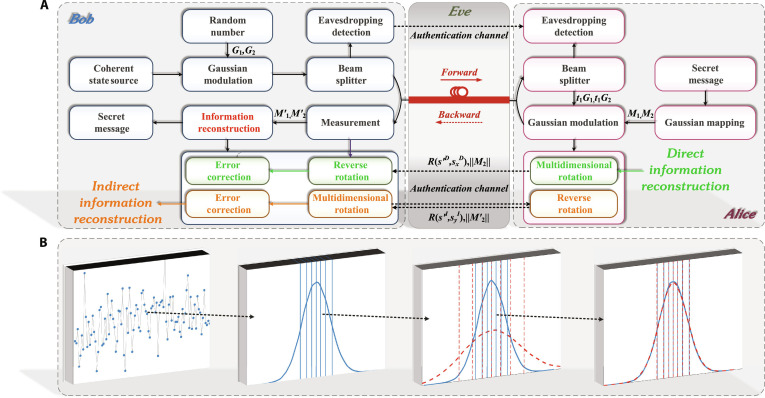
(A) Process diagram of direct information reconstruction and indirect information reconstruction scheme. *G*_1_, *G*_2_, 2 sets of Gaussian random numbers generated by Bob. $t_{1}$, a parameter determined by the transmittance of the forward channel. $M_{1}$, $M_{2}$, the Gaussian variables sent by Alice. $M_{1}^{\prime }$, $M_{2}^{\prime }$, the Gaussian variables received by Bob. $R\left({s^{\prime }}^{D},\;s_{x}^{D}\right)$, the rotation matrix of direct information reconstruction. $R\left({s^{\prime }}^{I},s_{x}^{I}\right)$, the rotation matrix of indirect information reconstruction. Dashed lines transmit classical information, while quantum information is sent via a thick purple solid line. (B) From left to right, the randomly generated initial Gaussian sequence, SC after Gaussian mapping (GM), SC after the quantum channel of both communication parties, and SC after information reconstruction. The solid line represents Alice’s SC and interval division, and the dashed line represents Bob’s.

In the information reconstruction stage, the communication parties use multidimensional rotation [57] based on the correlations among the Gaussian variables they hold, allowing them to recover the Gaussian variables of each other. Two schemes use different methods for recovering random numbers: direct and indirect reconstruction (as shown in Fig. 4A).

**Direct information reconstruction:** Bob recovers the Gaussian variables that carry the secret messages sent by Alice.

1. Symbol mapping: Alice performs multidimensional rotation on her Gaussian variables in the direct information reconstruction, as shown in Fig. 4A. Firstly, she performs symbol mapping on 1 of the 2 sequences. For example, Alice conducts symbol mapping on the $M_{1}$ sequence, mapping positive values to “0” and negative values to “1”, thus generating a set of binary sequences. Subsequently, he randomly selected a sequence $c^{D}$ (the superscript *D* represents the direct scheme) of length *K* from the binary sequence based on the error correction code rate $R_{c}$ ($R_{c}=K/N$, *N* is the number of subinformation blocks in an information block). Then, she encoded it as *s^D^* of length *N* using classical error correction codes.
2. Spherical mapping: Alice performs the spherical mapping on sequences $s^{D}$ and $M_{2}$, with a specified dimension of *d*. The specific mapping relationship is as follows
  $${s^{\prime }}^{D}=\left(s_{1}^{D},\;s_{2}^{D},\;\cdots ,\;s_{d}^{D}\right)\to \left(\frac{{\left(-1\right)}^{s_{1}^{D}}}{\sqrt{d}},\;\frac{{\left(-1\right)}^{s_{2}^{D}}}{\sqrt{d}},\;\cdots ,\;\frac{{\left(-1\right)}^{s_{d}^{D}}}{\sqrt{d}}\right)$$ (3)
  $$s_{x}^{D}=M_{2}/\left\|M_{2}\right\|=\frac{M_{2}}{\sqrt{\sum_{i=1}^{d}M_{2_{i}}^{2}}}$$ (4)
  Then, Alice calculates the rotation matrix $R\left({s^{\prime }}^{D},\;s_{x}^{D}\right)$ by combining the *d*-dimensional sphere sequences ${s^{\prime }}^{D}$ and normalized sequence $s_{x}^{D}$ after spherical mapping, which satisfies $R\left({s^{\prime }}^{D},\;s_{x}^{D}\right)s_{x}^{D}={s^{\prime }}^{D}$, i.e., $H\left({s^{\prime }}^{D}|s_{x}^{D},\;R\right)=0$. Bob sends the functions *R* and $\left\|M_{2}\right\|$ to Alice through the classical authentication channel.
3. Alice’s Gaussian variables reconstruction: Bob uses the received *R* and the local Gaussian variables $M_{2^{\prime }}$ to restore Alice’s Gaussian variables *M*_2_. The specific steps are as follows: Firstly, Bob performs the same rotation on the local sequence $M_{2^{\prime }}$, mapping it to the normalized sequence $s_{y}^{D}$ (similar to sequence *M*_2_). Then, he calculates the auxiliary sequence $v^{D}=R\left({s^{\prime }}^{D},\;s_{x}^{D}\right)s_{y}^{D}$ and decodes it, then performs spherical mapping to obtain the sequence ${s^{\prime }}^{D}$. Next, Bob reversely rotates $s_{y}^{D}$ to get the sequence $s_{x}^{D}$
  $$s_{x}^{D}=R{\left({s^{\prime }}^{D},\;s_{x}^{D}\right)}^{-1}{s^{\prime }}^{D}$$ (5)

In this way, Bob can calculate the Gaussian variables of Alice’s modulation information

$$M_{2}=\left\|M_{2}\right\|s_{x}^{D}$$ (6)

4. Error correction: Through the above calculation, when the auxiliary sequence $s^{D}$ is decoded correctly, Bob performs inverse GM on the obtained $M_{2}$ to get the secret messages. If $s^{D}$ decoding fails, this information block will be retransmitted.

**Indirect information reconstruction:** Alice recovers the Gaussian variables measured by Bob.

1. Symbol mapping: In the indirect reconstruction, as shown in Fig. 4A, Bob performs symbol mapping on 1 of the 2 sequences he holds $M_{1}^{\prime }$ and $M_{2}^{\prime }$, such as mapping $M_{1}^{\prime }$. Then, he randomly selects a portion of the binary sequence $c^{I}$ with a length of *K* from the mapped sequence and encodes $c^{I}$ into the sequence $s^{I}$ using a classical error correction code with code rate $R_{c}$ (the superscript *I* represents the indirect scheme).
2. Spherical mapping: Bob performs spherical mapping on sequence $s^{I}$ and $M_{2}^{\prime }$, with *d*-dimension, and the mapping relationship is similar to the direct scheme
  $${s^{\prime }}^{I}=\left(s_{1}^{I},\;s_{2}^{I},\;\cdots ,\;s_{d}^{I}\right)\to \left(\frac{{\left(-1\right)}^{s_{1}^{I}}}{\sqrt{d}},\;\frac{{\left(-1\right)}^{s_{2}^{I}}}{\sqrt{d}},\;\cdots ,\;\frac{{\left(-1\right)}^{s_{d}^{I}}}{\sqrt{d}}\right)$$ (7)
  $$s_{y}^{I}=M_{2}^{\prime }/\left\|M_{2}^{\prime }\right\|=\frac{M_{2}^{\prime }}{\sqrt{\sum_{i=1}^{d}M_{2i}^{\prime 2}}}$$ (8)
  Bob calculates the rotation matrix $R\left({s^{\prime }}^{I},\;s_{y}^{I}\right)$ using the sequence ${s^{\prime }}^{I}$ and $s_{y}^{I}$, which also satisfies $R\left({s^{\prime }}^{I},\;s_{y}^{I}\right)s_{y}^{I}={s^{\prime }}^{I}$. Bob sends *R* and $\left\|M_{2}^{\prime }\right\|$ to Alice through the classical authentication channel.
3. Bob’s Gaussian variables reconstruction: Alice uses the received *R* and the local Gaussian sequence $M_{2}$ to restore Bob’s Gaussian sequence $M_{2}^{\prime }$. The steps are as follows: Firstly, Alice performs the same rotation on the local sequence $M_{2}$, mapping it to the sequence $s_{x}^{I}$, and calculates the auxiliary sequence $v^{I}=R\left({s^{\prime }}^{I},\;s_{y}^{I}\right)s_{x}^{I}$. Decoding sequence $v^{I}$ and normalizing yields sequence ${s^{\prime }}^{I}$, which is then reverse rotated to obtain $s_{y}^{I}$
  $$s_{y}^{I}=R{\left({s^{\prime }}^{I},\;s_{y}^{I}\right)}^{-1}{s^{\prime }}^{I}$$ (9)
  In this way, Alice can calculate the Gaussian variables measured by Bob
  $$M_{2}^{\prime }=\left\|M_{2}^{\prime }\right\|s_{y}^{I}$$ (10)
4. Error correction: Through the above steps, when the auxiliary sequence $s^{I}$ is decoded correctly, Alice can obtain the correct $M_{2}^{\prime }$. By comparing the inverse GM intervals of 2 sets of Gaussian variables $M_{2}$ and $M_{2}^{\prime }$, it can be determined where Bob will obtain the error information after the inverse GM. Alice can notify Bob of the location of the error information through the classical authentication channel. Bob performs inverse GM on sequence $M_{2}^{\prime }$ to obtain subinformation blocks and then corrects based on the edge information notified by Alice, thereby obtaining the secret messages. If *s^I^* decoding fails, this information block will be retransmitted.

### System security analysis

**Fig. 5.**
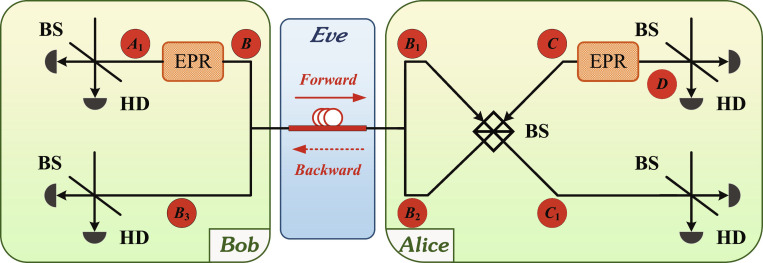
System diagram of entanglement-based model of continuous variable (CV) quantum secure communication (QSC) using coherent state. EPR, entangled source; BS, beam splitter; HD, heterodyne detection; $A_{1}∼D$, quantum states.

At the protocol implementation level, communication systems usually adopt the prepare-and-measure model for experimental implementation (as shown in Fig. 1A); in security analysis, the entanglement-based model is used (as shown in Fig. 5), but these 2 models have been proved to be mutually equivalent [58]. According to Wyner’s wiretap channel theory, the maximum information transmission capacity between Alice and Bob in a noisy environment can be modeled as the main channel $C_{M}$ [33]. Eve’s eavesdropping behavior and environmental noise are modeled by the eavesdropping channel $C_{W}$, representing the maximum amount of information Eve can extract from eavesdropping symbols without violating the basic principles of quantum mechanics. Wiretap channel theory suggests that when the secrecy capacity $C_{S}$ is greater than zero, both legitimate parties can ensure the transmission security of secret messages through some encoding method at a capacity lower than $C_{S}$ [32,33]. Essentially, secrecy capacity $C_{S}$ is the difference between the capacity of the main channel and the capacity of the wiretap channel, that is

$$C_{S}=C_{M}-C_{W}.$$ (11)

CV QSC’s core for calculating the secrecy capacity is the covariance matrix $\rho_{A_{1}B_{3}C_{1}D}$. The covariance matrix measured by Bob can be expressed as $\Gamma_{X_{1}X_{2}Y_{1}Y_{2}}=\frac{1}{2}\left(\rho_{A_{1}B_{3}C_{1}D}+I_{8}\right)$, where $X_{1}X_{2}Y_{1}Y_{2}$ are the classical information of the collapse of $A_{1}B_{3}C_{1}D$ after measurement. The main channel capacity *C_M_* is represented as

$$C_{M}=I\left(X_{2}:\left(Y_{1},\;Y_{2}\right)\right)={\text{log}}_{2}\frac{\text{det}\Gamma_{X_{2}}\text{det}\Gamma_{Y_{1}Y_{2}}}{\text{det}\Gamma_{X_{2}Y_{1}Y_{2}}}.$$ (12)

When the eavesdropper adopts collective attacks, it should be noted that the *C_W_* calculation for direct and indirect is different. In the direct scheme, the secret-message recovery relies on the multidimensional rotation of the data sent by Alice, hence $C_{W}=S_{Y_{2}E}$, the quantum mutual information between Alice and Eve. In the indirect scheme, secret-message recovery relies on the multidimensional rotation of data sent by Bob, hence $C_{W}=S_{X_{2}E}$, the quantum mutual information between Bob and Eve.

For the convenience of analyzing protocol security, when Bob initially prepares and distributes a quantum state with variance *V*, after Alice receives the quantum state but before measurement, the corresponding covariance matrix of the system’s state $\rho_{A_{1}B_{3}C_{1}D}$ is

$$\left[\begin{array}{cccc} V_{B}{𝕀}_{2} & * & * & \\ \sqrt{\tau T_{1}T_{2}\left(V_{B}^{2}-1\right)}\sigma_{Z} & \left[\tau T_{1}T_{2}\left(V_{B}+\chi_{\mathrm{line}1}\right)+\left(1-\tau \right)T_{2}V_{A}+T_{2}\chi_{\text{line}2}\right]{𝕀}_{2} & * & *\\ -\sqrt{\left(1-\tau \right)T_{1}\left(V_{B}^{2}-1\right)}\sigma_{Z} & -\sqrt{\tau \left(1-\tau \right)T_{2}}\left[T_{1}\left(V_{B}+\chi_{\text{line}1}\right)-V_{A}\right]{𝕀}_{2} & \left[\left(1-\tau \right)T_{1}\left(V_{B}+\chi_{\text{line}1}\right)+\tau V_{A}\right]{𝕀}_{2} & *\\ & \sqrt{\left(1-\tau \right)T_{2}\left(V_{A}^{2}-1\right)}\sigma_{Z} & \sqrt{\tau \left(V_{A}^{2}-1\right)}\sigma_{Z} & V_{A}{𝕀}_{2} \end{array}\right]$$ (13)

where *V_B_* is Bob’s modulation variance, $\chi_{\text{line}1}=1/T_{1}-1+\epsilon_{1}$ represents the linear noise, $T_{1}$ is the channel transmittance, *ϵ*_1_ is excess noise, and all are calculated with shot noise as a unit.

Taking direct information recovery as an example, *X*_2_ is the measurement result of $B_{3}$. The covariance matrix $\Gamma_{A_{1}C_{1}D|X_{2}}$ of state $A_{1}C_{1}D$ with measurement result *X*_2_ as a condition is calculated as follows

$$\Gamma_{A_{1}C_{1}D|X_{2}}=\Gamma_{A_{1}C_{1}D}-C_{A_{1}C_{1}{\mathrm{DX}}_{2}}^{T}{\left(\Gamma_{X_{2}}+I_{2}\right)}^{-1}C_{A_{1}C_{1}{\mathrm{DX}}_{2}}.$$ (14)

The submatrices of the above conditional covariance matrix (obtained by rearranging the rows and columns of $\rho_{A_{1}B_{3}C_{1}D}$) are as follows

$$\Gamma_{X_{2}}=\left[\tau T_{1}T_{2}\left(V_{B}+\chi_{\text{line}1}\right)+\left(1-\tau \right)T_{2}V_{A}+T_{2}\chi_{\text{line}2}\right]I_{2}$$ (15)

$$\begin{array}{c} \begin{array}{c} \Gamma_{A_{1}C_{1}D}=\left[\begin{array}{ccc} V_{B}I_{2} & * & \\ -\sqrt{\left(1-\tau \right)T_{1}\left(V_{B}^{2}-1\right)}\sigma_{Z} & \left[\left(1-\tau \right)T_{1}\left(V_{B}+\chi_{\text{line}1}\right)+\tau V_{A}\right]I_{2} & *\\ & \sqrt{\tau \left(V_{A}^{2}-1\right)}\sigma_{Z} & V_{A}I_{2} \end{array}\right] \end{array} \end{array}$$ (16)

$$\begin{array}{c} C_{A_{1}C_{1}DX_{2}}=\left[\sqrt{\tau T_{1}T_{2}\left(V_{B}^{2}-1\right)}\sigma_{Z}-\sqrt{\tau \left(1-\tau \right)T_{2}}\left[T_{1}\left(V_{B}+\chi_{\text{line}1}\right)-V_{A}\right]\right.\\ \left.I_{2}\sqrt{\left(1-\tau \right)T_{2}\left(V_{A}^{2}-1\right)}\sigma_{Z}\right] \end{array}$$ (17)

In the proposed scheme, the calculation of $S_{Y_{2}E}$ and $S_{X_{2}E}$ is limited by the Holevo bound

$$S_{Y_{2}E}=S\left(E\right)-S\left(E|m_{A}\right)\overset{\text{purification}}{\to }=S\left(A_{1}B_{3}C_{1}D\right)-S\left(A_{1}B_{3}C_{1}|D\right)$$ (18)

$$S_{X_{2}E}=S\left(E\right)-S\left(E|m_{B}\right)\overset{\text{purification}}{\to }=S\left(A_{1}B_{3}C_{1}D\right)-S\left(A_{1}C_{1}D|B_{3}\right)$$ (19)

where $S\left(\rho \right)$ is the von Neumann entropy of a quantum state and $S\left(E|m_{A\left(B\right)}\right)$ is the conditional entropy of the quantum state that Eve grasps after knowing Alice’s (Bob’s) measurement results. Since $S\left(E\right)=S\left(A_{1}B_{3}C_{1}D\right)$ is the von Neumann entropy of the system, it is independent of the choice of scheme, and therefore, it is the same for both direct and indirect schemes. According to Williamson’s theorem, the calculation of the Gaussian state von Neumann entropy can be simplified by the symplectic eigenvalues of the covariance matrix

$$S_{Y_{2}E}=\sum_{i=1}^{4}G\left(\lambda_{i}\right)-\sum_{i=1}^{3}G\left(\lambda_{i}^{D}\right)$$ (20)

$$S_{X_{2}E}=\sum_{i=1}^{4}G\left(\lambda_{i}\right)-\sum_{i=1}^{3}G\left(\lambda_{i}^{I}\right)$$ (21)

where the function $G\left(x\right)$ is defined as

$$G\left(x\right)≔\frac{x+1}{2}{\text{log}}_{2}\left(\frac{x+1}{2}\right)-\frac{x-1}{2}{\text{log}}_{2}\left(\frac{x-1}{2}\right).$$ (22)

The following symplectic invariants $\Delta_{m,k}$ must first be established to determine the symplectic eigenvalues of the covariance matrix $\Gamma_{A_{1}B_{3}C_{1}D}$ [59].

$$\Delta_{m,k}=M_{2k}\left(i{\Omega \Gamma }_{A_{1}B_{3}C_{1}D}\right),$$ (23)

where *m* is the number of modes, $\Omega ={⊕}_{i=1}^{m}\left[\begin{array}{cc} 0 & 1\\ -1 & 0 \end{array}\right]$. $M_{2k}\left(i{\Omega \Gamma }_{A_{1}B_{3}C_{1}D}\right)$ is the *2k*-order principal minor of the 2m×2m matrix ${\Omega \Gamma }_{A_{1}B_{3}C_{1}D}$, that is, the sum of the determinants of all 2k×2k submatrices of ${\Omega \Gamma }_{A_{1}B_{3}C_{1}D}$. Then, the symplectic eigenvalues can be used to solve the polynomial of the following 4 modes, 3 modes, and 2 modes

$$z^{4}-\Delta_{4,1}z^{3}+\Delta_{4,2}z^{2}-\Delta_{4,3}z+\Delta_{4,4}=0$$ (24)

$$z^{3}-\Delta_{3,1}z^{2}+\Delta_{3,2}z-\Delta_{3,3}=0.$$ (25)

Specifically, we noticed that although information recovery can extract secret messages, it provides Eve with additional information. Therefore, the secrecy capacity after information recovery should be rewritten as

$$C_{S}^{D}=I_{\mathrm{AB}}-S\left(M_{2}:E,\;R\right)$$ (26)

$$C_{S}^{I}=I_{\mathrm{AB}}-S\left(M_{2}^{\prime }:E,\;R\right)$$ (27)

Alice and Bob can estimate the upper bound of $S_{Y_{2}E}$ and $S_{X_{2}E}$ on the basis of their covariance matrix. However, $S\left(M_{2}:E,\;R\right)$ and $S\left(M_{2}^{\prime }:E,\;R\right)$ cannot be inferred from them. Fortunately, we have the following theorem.

**Theorem:** Let A and B be classical random variables, and let E be a random quantum state. If A and B are independent of each other, then $S\left(A:E,B\right)\leq S\left(A,B:E\right)$ [57].

Next, according to the chain rule of mutual quantum information

$$S\left(M_{2},\;s_{x}^{D}:E\right)=S\left(M_{2}:E\right)+S\left(s_{x}^{D}:E|M_{2}\right).$$ (28)

Since $s_{x}^{D}$ is a symbol mapping of $M_{1}$’s random sequence and then encoded, independently of $M_{2}$, this means that $S\left(s_{x}^{D}:E|M_{2}\right)=0$, $S\left(M_{2},\;s_{x}^{D}:E\right)=S\left(M_{2}:E\right)=S_{Y_{2}E}$ . Coincidentally, in information recovery, $M_{2}$ and *R* are independent of each other, so there is $S\left(M_{2}:E,\;R\right)\leq S\left(M_{2},\;R:E\right)$. Then, since *R* is a function of $M_{2}$ and $s_{x}^{D}$, $\begin{array}{c} \begin{array}{c} S\left(M_{2}:E,\;R\right)\leq S\left(M_{2},\;R:E\right)=S\left(M_{2}:E\right)=S_{Y_{2}E} \end{array} \end{array}$. $S\left(M_{2}^{\prime }:E,\;R\right)$ can also derive $S\left(M_{2}^{\prime }:E,\;R\right)\leq S_{X_{2}E}$. Then, the lower bound of secrecy capacity after information recovery remains:

$$C_{S}^{D}=I_{\mathrm{AB}}-S\left(M_{2}:E,\;R\right)\geq I_{\mathrm{AB}}-S_{Y_{2}E}$$ (29)

$$C_{S}^{I}=I_{\mathrm{AB}}-S\left(M_{2}^{\prime }:E,\;R\right)\geq I_{\mathrm{AB}}-S_{X_{2}E}$$ (30)

In addition, due to the possibility of errors during the decoding process, the recovery of random numbers is not accurate, manifested as an IBER in the information recovery. Since we are calculating the secrecy capacity of the backward channel, a coefficient of $\frac{1}{2}$ needs to be introduced. Given this, the final expression for the secrecy capacity is

$$C_{S}^{D}=\frac{1}{2}\left(\left(1-\text{IBER}\right)I_{\mathrm{AB}}-S_{Y_{2}E}\right)$$ (31)

$$C_{S}^{I}=\frac{1}{2}\left(\left(1-\text{IBER}\right)I_{\mathrm{AB}}-S_{X_{2}E}\right)$$ (32)

where IBER is the ratio of erroneous information blocks to the total number.

## Data Availability

The materials and data can be obtained from the corresponding author on reasonable request.

## Supplementary Materials

Supplementary 1 Figs. S1 to S4 References [60,61]
