# Supplementary material for: Realization of High-Reliable Coherent-State Quantum Secure Communication
Source: Research (Wash D C). 2026 Jun 1;9:1290. doi: 10.34133/research.1290 (PMC13224318; doi:10.34133/research.1290)
Supplement: Supplementary 1 — Figs. S1 to S4 References [60,61] [file research.1290.f1.pdf]

Realization of High-reliable Coherent-State Quantum Secure Communication

Xinlei Chen[1†], Geng Chai[1†], Lei Wang[1], Sijie Wang[1], Xiaojie Chen[1], and Zhengwen Cao[1*]

[1]Laboratory of Quantum Information and Technology, School of Electronic Information, Northwest University, Xi'an 710127, China.
[*]Address correspondence to: caozhw@nwu.edu.cn
[†]These authors contributed equally to this work.

Supplementary Materials

1.1 Actual performance of the Gaussian mapping

The CV QSC, based on GM, provides a specific method to mapping secret messages to Gaussian random numbers. Specifically, GM first divides the secret message into blocks and then applies code conversion to make the probabilities of "0" and "1" in the secret message the same. Use the replaced codeword as sub-information blocks in the initial information block, and perform uniformization of the sub-information blocks while still using code conversion to complete. Insert random detection bits and error correction bits for each sub-information block. Finally, according to different mapping rules, each sub-information block will be mapped to any Gaussian random number in different partitioned regions.

GM connects secret messages with Gaussian modulation to ensure the secure transmission of secret messages. However, in practical environments, quantum state transmission and measurement are inevitably affected by the unknown characteristics of quantum channels and imperfect practical application equipment. Additionally, as the transmission distance increases, the channel loss also doubles. In this channel environment, a severe deviation exists between the Gaussian random number characteristics of the secret messages carried by the legitimate sender, Alice, and the Gaussian random number characteristics received by the legitimate receiver, Bob. Although the GM interval has a certain tolerance for noise, as this deviation increases, the system's tolerance will gradually decrease, thereby affecting the overall performance of the system and the accuracy of secret messages. Fig. S1 compares the SC and interval partitioning of the Gaussian random numbers Alice sent and Bob received under the same GM rule. From left to right are the comparisons of SC of SNR 5 dB, 0 dB, and -10 dB. From Fig. S1, we observe that when the SNR is high, the SC of the data sent

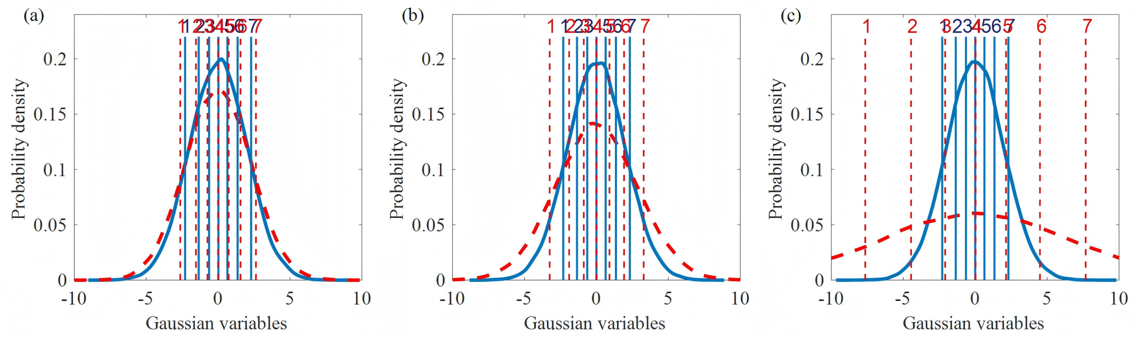

Fig. S1. Comparison of SC and interval partitioning of Gaussian variables Alice sent and Bob received when applying the same GM rule under different signal-to-noise ratios (SNRs). The horizontal axis represents Gaussian variables and is divided into eight intervals with equal probability, and the vertical axis represents probability density. The blue solid line in the figure represents Alice's side, the red dashed line represents Bob's side, and the numbers in the lines represent the boundaries of the interval division. The simulated SNR ratios from left to right are 5 dB, 0 dB, and −10 dB, respectively.

by Alice closely resemble those received by Bob, and the boundaries of the interval division slightly overlap. Consequently, the accuracy of Bob's secret message recovery is high. However, the SNR decreases as the communication distance increases or channel noise and interference intensify. In such cases, the SC of the data sent by Alice increasingly diverge from those received by Bob, causing the interval division boundaries to be more widely separated. According to the GM principle, this naturally leads to a higher error rate for Bob when reconstructing the secret messages. Therefore, we propose an information recovery in the original text to solve the above problems.

1.2 Self-balanced homodyne detector

1.2.1 Hardware design of the self-balanced homodyne detector

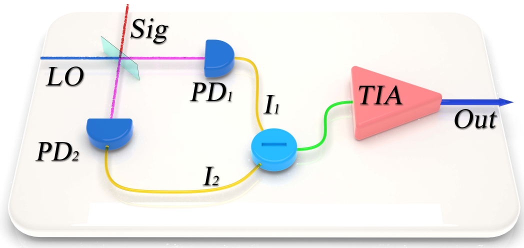

Fig. S2. Homodyne detector schematic. LO, local oscillator light; Sig, signal light; PD, photodiode; TIA, trans-impedance amplifier.

The homodyne detector uses a 50:50 beam splitter (BS) to couple the signal light (Sig) and the local oscillator light (LO). Then, two photodiodes (PD) convert the interfered light signal into photocurrent and perform differential amplification to obtain a differential electrical signal. Its typical structure is shown in Fig. S2. Due to the unbalanced BS splitting ratio, optical path

loss, photodiode sensitivity, etc., it is impossible to have a completely balanced homodyne detector (BHD). Assuming that the actual splitting ratio of BS is $m : (1-m)$, where $m \in (0,1)$; the optical path losses are L_1 and L_2, respectively; the sensitivities of the two photodiodes are R_{pin1} and R_{pin2}, respectively. Then their photoelectric conversion efficiencies are $\eta_{1,2} = R_{pin1,2} \cdot \frac{\hbar\nu}{e}$, where e is elementary charge, $\hbar$ is planck constant, ν is the frequency of light. The photocurrents of the two photodiodes are

$$I_1 = R_{pin1} \cdot m \cdot L_1 \cdot \frac{\hbar\nu}{\tau} \cdot \left(\hat{a}_s^\dagger + e^{-i\theta}\hat{a}_{LO}^\dagger\right)\left(\hat{a}_s + e^{i\theta}\hat{a}_{LO}\right) \tag{1}$$

$$I_2 = R_{pin2} \cdot (1-m) \cdot L_2 \cdot \frac{\hbar\nu}{\tau} \cdot \left(\hat{a}_s^\dagger - e^{-i\theta}\hat{a}_{LO}^\dagger\right)\left(\hat{a}_s - e^{i\theta}\hat{a}_{LO}\right), \tag{2}$$

where τ is the duration of the optical pulse. With the transimpedance amplification gain of the BHD being G, its output voltage is

$$U = G\left(I_1 - I_2\right) = G \cdot R_{pin} \cdot \frac{\hbar\nu}{\tau}\left(\hat{a}_s^\dagger\hat{a}_{LO}e^{i\theta} + \hat{a}_{LO}^\dagger\hat{a}_s e^{-i\theta}\right). \tag{3}$$

We can see that the diode's sensitivity, the BS's splitting ratio, and optical path loss similarly affect the photocurrent. Therefore, all the effects can be equivalent to the sensitivity of the photodiode, and the equivalent diode sensitivities R_1 and R_2 are satisfied

$$R_1 = 2 \cdot R_{pin1} \cdot m \cdot L_1 \tag{4}$$

$$R_2 = 2 \cdot R_{pin2} \cdot (1-m) \cdot L_2 \tag{5}$$

Then the output voltage of the detector is the following

$$\begin{aligned} U' &= G \cdot \frac{\hbar\nu}{2\tau} \cdot \left[(R_1 - R_2)\left(\hat{a}_s\hat{a}_s^\dagger + \hat{a}_{LO}\hat{a}_{LO}^\dagger\right) + (R_1 + R_2)\left(\hat{a}_s\hat{a}_{LO}^\dagger e^{-i\theta} + \hat{a}_s\hat{a}_{LO}^\dagger e^{i\theta}\right)\right] \\ &= G \cdot \frac{\hbar\nu}{2\tau} \cdot \left[(R_1 - R_2)\left(\langle\hat{n}_s\rangle + \langle\hat{n}_{LO}\rangle\right) + (R_1 + R_2)\left(\hat{a}_s\hat{a}_{LO}^\dagger e^{-i\theta} + \hat{a}_s\hat{a}_{LO}^\dagger e^{i\theta}\right)\right], \end{aligned} \tag{6}$$

where $\langle\hat{n}_s\rangle = \hat{a}_s\hat{a}_s^\dagger$, $\langle\hat{n}_{LO}\rangle = \hat{a}_{LO}\hat{a}_{LO}^\dagger$. Compared with Eq. (3), it is observed that additional detection noise is introduced in the unbalanced optical path, and this type of noise is referred to as common-mode noise U_{cmn}, expressed as

$$U_{cmn} = G \cdot \frac{\hbar\nu}{2\tau} \cdot (R_1 - R_2)\left(\langle\hat{n}_s\rangle + \langle\hat{n}_{LO}\rangle\right), \tag{7}$$

and the variance of the noise is

$$Var\left(U_{cmn}\right) = G^2 \cdot \left(\frac{\hbar\nu}{2\tau}\right)^2 \cdot (R_1 - R_2)^2 \cdot \left[Var\left[\langle\hat{n}_s\rangle\right] + Var\left[\langle\hat{n}_{LO}\rangle\right]\right], \tag{8}$$

where $Var\left[\langle\hat{n}_s\rangle\right] = \left(\frac{V_M^2}{4}\right)$ and $Var\left[\langle\hat{n}_{LO}\rangle\right] = \left(\frac{\tau}{\hbar\nu}\right)^2\langle P_{LO}\rangle^2$. An unbalanced homodyne detector's common-mode noise variance is proportional to the square of the unbalance coefficient $(R_1 - R_2)$. As a result, this imbalance introduces additional noise and depletes communication performance.

To minimize the impact of optical path mismatch, the sensitivity of many photodiodes can be

tested to screen out the pair with the most compatible performance [1]. However, this method cannot fundamentally solve the problem of optical path asymmetry. Another solution is to connect two variable optical attenuators on the optical paths between the BS and photodiode, respectively, and compensate for the mismatch problem by dynamically adjusting the attenuation ratio. However, this compensation method will introduce additional optical loss, decreasing detection efficiency and reducing system performance [2].

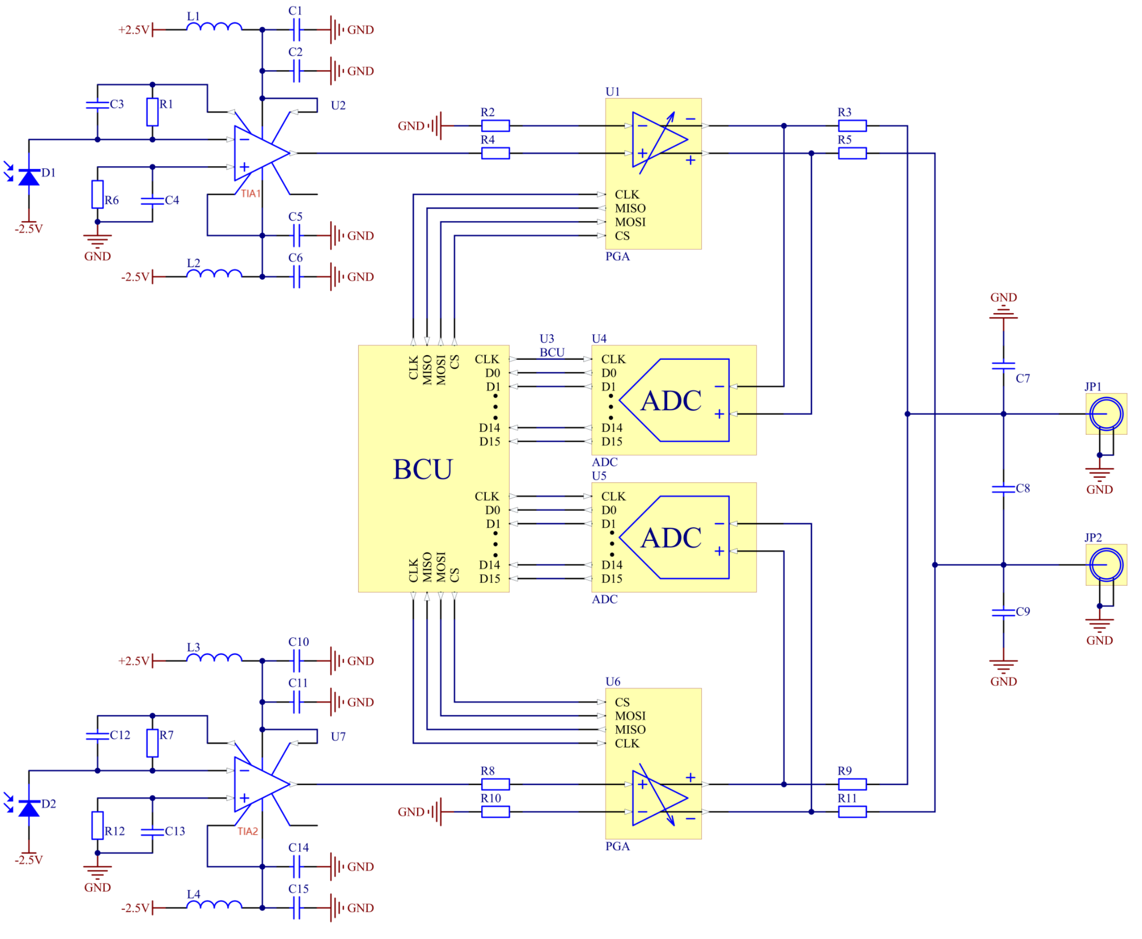

Fig. S3. The simplified hardware schematic. BCU, balance control unit; ADC, analog-to-digital converter; PGA, programmable gain amplifier; TIA, trans-impedance amplifier; D, photodiode; R, resistor; C, capacitor.

We propose a self-BHD based on a programmable gain amplifier (PGA) to address the issues above. The simplified hardware schematic of the designed self-BHD is shown in Fig. S3, which can automatically detect the two input unbalanced optical paths and calibrate them automatically. Specifically, the photocurrents generated by the two photodiodes are amplified by two trans-impedance amplifiers (TIA) and amplified by a first-stage PGA. A differential voltage signal is generated through an analog subtractor and then output by a low-pass filter (LPF). At the same time, the two PGA output signals are sampled by analog-to-digital converters (ADCs), and the balance

control unit (BCU) calculates the unbalanced signal ratio and adjusts the PGA gain to compensate for the unbalanced system. The BCU can also detect the output signal amplitude and saturation phenomenon to realize the adaptive gain control (AGC) function. When the output is saturated, the gain of the self-BHD is automatically reduced to ensure the signal's integrity. Conversely, suppose the signal amplitude is too small. In that case, the gain of the self-BHD is automatically increased, effectively improving the SNR of small signals and the detector's dynamic range.

Assuming that the product of the gain of two PGA channels and the gain of the TIA amplifier is G_1 and G_2, respectively, according to Eq. (2), the output voltages U_1 and U_2 of the two PGA channels are:

$$U_1 = G_1 I_1 = G_1 R_1 \cdot \frac{\hbar\nu}{2\tau} \cdot \left(\hat{a}_s^\dagger + e^{-i\theta}\hat{a}_{LO}^\dagger\right)\left(\hat{a}_s + e^{i\theta}\hat{a}_{LO}\right) \tag{9}$$

$$U_2 = G_2 I_2 = G_2 R_2 \cdot \frac{\hbar\nu}{2\tau} \cdot \left(\hat{a}_s^\dagger - e^{-i\theta}\hat{a}_{LO}^\dagger\right)\left(\hat{a}_s - e^{i\theta}\hat{a}_{LO}\right) \tag{10}$$

The final output voltage U_0 of the self-BHD is:

$$U_0 = \frac{U_1 - U_2}{2} = \frac{\hbar\nu}{4\tau}\left[(G_1R_1 + G_2R_2)\left(e^{i\theta}\hat{a}_s^\dagger\hat{a}_{LO} + e^{-i\theta}\hat{a}_{LO}^\dagger\hat{a}_s\right) + (G_1R_1 - G_2R_2)\left(\hat{a}_s^\dagger\hat{a}_s + \hat{a}_{LO}^\dagger\hat{a}_{LO}\right)\right] \tag{11}$$

If $(G_1R_1 - G_2R_2)$ is satisfied, the common-mode term of the output voltage U_0 is zero, that is

$$U_0 = \frac{\hbar\nu}{4\tau}(G_1R_1 + G_2R_2)\left(e^{i\theta}\hat{a}_s^\dagger\hat{a}_{LO} + e^{-i\theta}\hat{a}_{LO}^\dagger\hat{a}_s\right). \tag{12}$$

Therefore, by setting the gain of the PGA to meet

$$\frac{G_1}{G_2} = \frac{R_2}{R_1} = \frac{R_{pin2}\cdot(1-m)\cdot L_2}{R_{pin1}\cdot m\cdot L_1}, \tag{13}$$

the asymmetry of the optical path can be compensated for, thereby eliminating the common-mode noise caused by unbalanced optical paths.

1.2.2 Self-balanced homodyne detector performance test

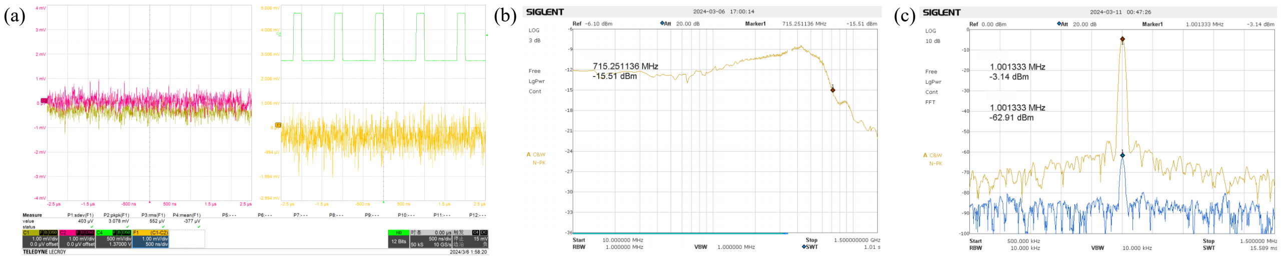

Fig. S4. The test results of relevant indicators of the self-BHD. (a) Electrical noise test results of self-BHD. (b) Frequency response curve of self-BHD. (c) The test results of relevant indicators of the self-BHD.

We test three detector indicators: electronic noise, bandwidth, and common-mode rejection

ratio. The electronic noise is an essential indicator of the detector, and excessive noise will impact the output accuracy of the signal detection. As shown in Fig. S4 (a), C1 is the in-phase output signal of the detector, C2 is the inverted output signal, and C4 is the modulation waveform of AM. Since the self-BHD uses a differential voltage as its output, the detector's effective signal output is the difference between the above two waveforms, i.e., F1. The test results show that the electronic noise variance of the detector is 1.624×10^{-7} V^2. The bandwidth of a detector is one of the most critical indicators of a detector, reflecting the frequency range of the detected signals. The frequency response of the self-BHD is characterized in Fig. S4 (b). The tested results show that the bandwidth of the self-BHD is about 715 MHz, with the gain fluctuation in the passband less than 3 dB. The common-mode rejection ratio is another important indicator of the detector, reflecting the resistance to common-mode interference and the gain for differential signals. The spectrum analyzer is employed to test the spectrum of the output signals of the self-BHD under differential mode input and common-mode input, which is presented in Fig. S4 (c). The upper yellow curve shows the output signal spectrum of the self-BHD at differential mode input, and its output fundamental frequency signal strength is -3.14 dBm. The blue curve below shows the output signal spectrum of the self-BHD with a common-mode input, and its output fundamental frequency signal strength is -62.91 dBm. Therefore, the common-mode rejection ratio of the self-BHD is 59.77 dBm. In addition, due to the photodiode responsivity being 0.95 and the insertion loss of the BS four ports being 0.9483, the total detection efficiency of self-BHD is $76.19\% \times 94.83\% = 72.25\%$. The advantage of this self-BHD is that it achieves high-bandwidth automatic balance control without modifying the optical path or adding optical devices.

References

1. Cooper M, Söller C, and Smith BJ. High-stability time-domain balanced homodyne detector for ultrafast optical pulse applications. Journal of Modern Optics 2013;60:611–6.

2. Zou M, Mao Y, and Chen TY. Rigorous calibration of homodyne detection efficiency for continuous-variable quantum key distribution. Optics Express 2022;30:22788–97.
